# Setting Primary Health and Social Care Priorities Using a Deliberative Democratic Participatory Approach

**DOI:** 10.1111/hex.70173

**Published:** 2025-02-04

**Authors:** Coralie Wales, Penny Abbott, Jackie Street, Lauren Fawcett, Maree Jennings, Wendy Sharp, Philip Lee

**Affiliations:** ^1^ Western Sydney University Penrith New South Wales Australia; ^2^ Lead Facilitator Community Partnerships WentWest Westmead New South Wales Australia; ^3^ School of Medicine Western Sydney University Penrith New South Wales Australia; ^4^ School of Public Health University of Adelaide Adelaide South Australia Australia; ^5^ Relationships to Partnerships Practitioner WentWest Westmead New South Wales Australia; ^6^ First Nations Community Facilitator Westmead New South Wales Australia; ^7^ Volunteer Consumer Advisor WentWest Westmead New South Wales Australia

**Keywords:** citizen's jury, deliberative methods, primary health, social determinants of health

## Abstract

**Introduction:**

Publicly funded regional primary health care investments are usually informed by analysis of locally collected data, surveys, focus groups and consultations. We wanted a deeper understanding of baseline health and social care priorities in culturally diverse Western Sydney. If WentWest could understand the priorities, the operational agenda for commissioning services could be tailored around them. This research describes the organisation, conduct and recommendations of a citizens' jury in 2023.

**Methods:**

Thematic analysis of the transcribed facilitated discussions identified the well‐informed priorities of a diverse and representative group of Western Sydney citizens. Listening to internal and external advisory groups delivered communication pathways, expert presenters, handbook preparation and practical support for the jury event. Consistent with the principle that research about the community should include the community, four consumer advisors were colleagues in the research team.

**Results:**

The jury supported a focus on prevention, including early intervention to keep children thriving and connected at school, and better cross‐sectoral bonds via coalitions to enable social care to be linked to health care. Team‐based care in general practice for proactive data use and building a culture for relationship‐based care, developing a patient‐centred workforce through incentivising students and expediting registration of overseas trained health workers were recommended. Assets‐based community development was seen as an essential approach in linking the community with these recommendations.

**Conclusion:**

The use of citizens' juries in primary health settings is rare. In this culturally diverse community, WentWest now has a foundational understanding of what investments will deliver and has committed to institutionalising this deliberative approach into a permanent citizens assembly.

**Patient and Public Contribution:**

The design of the jury was guided by several advisory groups across the local region. The research team included four consumer advisors to provide overarching feedback and external insights. The jurors were selected to be representative of the regional community. They developed the recommendations reported in this article.

**Trial Registration:**

This research does not involve clinical trials.

## 1 Introduction

Building strong relationships with communities to enable a deep understanding of the health and social care priorities of the population is crucial in determining effective investments in regional services. Data‐driven analyses and periodic surveys are often distributed across the region, and other traditional methods like focus group activities are used to inform what is needed in a region. This article discusses a more ambitious approach involving deliberative democratic action in the primary health landscape.

WentWest has been part of the Western Sydney Community for 20 years and in 2015, took on the major role of the Western Sydney Primary Health Network (WSPHN) responsible for serving the local government areas (LGAs) of Western Sydney. Primary Health Networks (PHNs) are a Federal Government–funded Australian health initiative that aims to ‘streamline health services, particularly for those at risk of poor health outcomes’ and to better coordinate care so that ‘people receive the right care in the right place at the right time’ [1]. PHNs are required to assess local health needs, commission required health services, help build appropriate health workforce capacity and connect health services to ‘encourage better use of health resources and avoid duplication’ [2]. In pursuing these goals, WSPHN builds relationships with local general practitioners, allied health professionals, the community and the hospital system. WSPHN supports more than 6000 medical, allied health, nursing, pharmacy and support staff. It also supports 60 residential aged care facilities [3].

WSPHN serves one of Australia's largest economic regions and one of the fastest‐growing population and employment centres. It is a richly multicultural community with people from over 170 different countries, a large Indigenous population and a high proportion of persons who arrived as refugees. The Western Sydney area is part of Greater Western Sydney—a larger region that extends to include 13 LGAs. By 2031, Greater Western Sydney will accommodate over half of Sydney's population [4]. Similar to many Australian cities, there has been inadequate local infrastructure investment, housing affordability has declined sharply and extreme urban heat threatens [5].

Compared to national incidence figures, the population served by WSPHN has more than twice the rate of diagnosed diabetes (12% vs. 5% Australia [6, 7] and significantly lower life expectancy compared to other LGAs [8]. Residents of the Western Sydney region are much less likely to have seen a dental professional in the past year (45.8% vs. 63.6% in Northern Sydney [9]).

To understand and address these challenges, WSPHN has been engaging for many years with community organisations and individuals in multiple ways including a permanent Consumer Advisory Council [10]. The objective has been to bring community perspectives and preferences to the centre of the organisation's planning and service delivery. Although the council consists of a range of community/organisational leaders, consumer representatives and advocates, WSPHN acknowledged that their understanding of community priorities could be deepened. During council meetings, consideration of the needs of Western Sydney communities was primarily limited to commentary about existing programs rather than accessing insights about community priorities.

In 2023, WentWest committed to the use of a diverse, participatory and informed engagement exercise grounded in deliberative democratic theory and based on the citizens' jury model [11, 12]. Since 2021, WentWest has focused on participatory methods, adopting a co‐design approach with the Consumer Advisory Council and including consumer involvement in research teams within the Community Development portfolio.

In 2023, we brought together a group of people who would demographically reflect the diversity of the Western Sydney community to consider a range of evidence about how health and social care provision operated in Western Sydney and beyond. The participants were asked to deliberate in a facilitated and respectful environment to provide recommendations for the direction and priorities of the community. A separate jury, described elsewhere, engaged the Western Sydney First Nations community.

This article reports the process and results of a culturally diverse citizens' jury. ‘Culturally diverse’ in this context includes all migrant populations whose ancestors arrived in Australia in 1788 or later. The citizens' jury was asked to consider the following question:

‘Should we, the people of Western Sydney continue to invest in the health system in the same way as we have in the past?’

## 2 Methods

The citizens' jury approach allows a variably informed group to rapidly reach a level of understanding of the relevant social and ethical factors and underpinning evidence, to allow them to engage effectively on a complex policy question. Diversity of views is represented both through the appropriate recruitment of a diverse jury of people and in the presentation of evidence to the jury (see Table 3). The goal of this study is to reason together for the public good [13] and to generate recommendations to guide policy.

The proceedings were audio‐recorded and transcribed by a Hansard reporter who identified the speakers. The transcripts were analysed using realist thematic analysis [14]. One author who had not attended the jury extracted the major themes from the data, as they elucidated the reasons for the jury's recommendations. Using NVivo, a second author who had been present at the jury independently coded the jury's deliberations using some pre‐established codes and identifying other codes as they emerged. Individual codes were grouped thematically to provide patterns of views that account for the final priority recommendations. The two forms of analysis demonstrated high agreement.

### 2.1 Consumer Participation in the Research Team

The active involvement of consumers in health research aligns with the principle that those who are affected by research have a right to inclusion in what and how research is undertaken [15]. Inclusion adds value by grounding the research in what matters most to consumers [16] and builds capability and confidence in consumers as research colleagues. In planning this research, we included four consumer advisors (Table 1) as colleagues in our research team, to provide overarching feedback and external insights.

**Table 1:** Consumer advisors.

CA1	Local retired health practitioner volunteer as a consumer advisor for the project
CA2	Local First Nations person involved as facilitator in separate jury in Western Sydney (not reported in this article)
CA3	Local retired health practitioner consumer advisor with experience in previous consumer research in the local area [16]
CA4	Local First Nations person involved in providing advice for the second jury (not reported in this article)

Abbreviation: CA = consumer advisor.

### 2.2 Advisory Inputs

Preparation for the jury was guided by several cross‐sectoral advisory groups drawn from internal and external Western Sydney partnerships. Advisory members were chosen to reflect a diverse range of community, professional, academic and lived experience (Table 2).

**Table 2:** Advisory groups.

	Group	Members	Meetings 2023
1	Consumer Advisory Council	Community and Clinical Leaders living and working in Western Sydney. Includes professional leaders from general practice, allied health, oral health, community health, community developers, people with lived experience of chronic health conditions, advocates in cultural and linguistic diversity and health advocates for early and later years.	5 × 1 h February to November
	Clinical Council		
2	WentWest internal advisors	Convenience sample WentWest colleagues interested in citizens' jury approach and able to attend.	3 × 90 min February to May
3	External advisory group	Eight purposively selected individuals—insights into views of Western Sydney Local Government, NSW Health, health consumers, refugees, new migrants, people with chronic diseases, young people and older people.	3 × 90 min February to May
4	Individual consultations	WentWest program leaders—advise how to connect to the internal program agenda. A two‐way process to ensure WentWest was able to ‘own’ process and outcome.	8 × 1 h February to December and ongoing
5	Community Leaders Forum	Forum: 21 invited leaders—introduce the concept of a citizens' jury and seek advice on content, structure and language.	May

The WentWest Consumer and Clinical Advisory Councils (‘the Councils’) provided significant oversight. We sought further advice from purposively selected groups with additional expertise. Two advisory groups met for 90 min three times, whereas the Councils met several times before and after the citizens' jury was convened. Discussions focused on how specific community groups would best receive complex information, choice of evidence and experts, and how health systems work for sections of the community. Some advisors provided practical support during the jury (field notes, organising and recording discussion). A counsellor was present at the jury for anyone who needed support.

Explicit advice was that the evidence should avoid complex statistical information that might obscure the most relevant, illustrative and informative data. Advisors strongly recommended that evidence should use video and graphic representation, and the evidence would have more meaning using a life‐course model following the lives of two hypothetical Western Sydney families.

### 2.3 Selection of Experts

To present the evidence, experts (*N* = 20) were selected (Table 6).

Evidence addressed major areas of need in the local community as informed by previous needs assessments by the PHN. Local cultural diversity and levels of socioeconomic disadvantage guided what evidence was included. Sources of evidence included local public health collected data and other high‐quality government and peer‐reviewed published information.

Experts were advised not to use slides, but rather, to use a conversational approach, illustrating their points by reference to two hypothetical children introduced in the handbook. Experts were invited to submit material for inclusion in the handbook. Following the presentations, equal time was allowed for questions and discussion. A complete description of the presenters, presentations and sources of evidence can be found in the jury handbook (available here). The handbook was provided to the jury 2 weeks before the jury, and participants were asked to review it before the jury.

### 2.4 Recruitment of Participants

Demographic factors commonly used to guide recruitment of citizens' juries were used, including a spread of age, gender, education, employment, postcode and cultural background, deliberately reflecting the population profile of people in Western Sydney. These criteria are in line with the standard citizens' jury process. The cultural background was defined by cultural ancestry and religion. The aim was to provide a diverse group that would encapsulate a broad range of the experiences and preferences of the Western Sydney community. Recruitment was conducted by CRNRSTONE Research from their national online opt‐in database of participants [17]. Participants were provided an honorarium of $400 AUD. Two additional participants were on standby in the event of participant drop‐out.

Participants were eligible if they were over 18 years of age, available on the dates of the jury, lived in Western Sydney, consented to participate, could communicate in English and were connected to the Internet (to review the handbook).

The jury convened over a weekend in August 2023. On Friday night, participants shared a meal and learned about the program. At this meeting, the WentWest CEO assured participants that their recommendations would form the WSPHN agenda for short, medium or longer term implementation, and cross‐sector recommendations would be shared with other Western Sydney parties for coalition action. On Saturday, the jury heard the evidence and on Sunday they deliberated and made recommendations.

On day 3, the CEO again committed to the recommendations and confirmed that reports would be made to the jury, WentWest, the Western Sydney community and a peer‐reviewed journal.

### 2.5 Role and Experience of Facilitator

High‐quality, expert facilitation is linked to the extent to which deliberation succeeds in producing considered views [18]. An expert facilitator builds skills and confidence [19] and supports groups as they make sense of complex information [20]. The professional facilitator (C.W.) had over 20 years of experience facilitating a range of groups in discovery and co‐design environments, as well as experience facilitating two local juries [21]. The facilitator is the lead author and investigator.

Reporting of the work follows the CJ (citizens' jury) Check framework [22]. The research was approved by the Western Sydney University Human Research Ethics Committee (HREC approval H15450).

## 3 Results

### 3.1 Participants

Twenty people attended, but two participants were unable to attend, so both standby participants were included (Table 3).

**Table 3:** Citizen jury demographics.

*Gender*: 8 female, 11 male, 1 nonbinary *Age* (reflecting the younger demographic of Western Sydney): 18–35 = 8 36–55 = 7; and 56+ years = 5 *Types of employment*: Full‐time = 10; part‐time or casual = 3, retired = 1, pension = 1, student not working = 1, student working = 2, home duties = 2 *Personal income*: 5 below $45,214/year (median income for Western Sydney) (Table 4) *Highest educational attainment*: High school = 4, Technical/Trade Certificate = 6, university undergraduate degree = 5, university postgraduate degree = 4 *Chronic health condition*: 6/20 *Ethnicity/cultural background*: Anglo (Australian, British and Irish) = 5, European = 2, Indian = 6, Filipino = 3, Chinese = 2, Middle Eastern = 3, Arabic = 1, South Korean = 1 (Note: Three participants claimed two ethnicities) *Religion*: Catholic = 8, Christian = 3, Islam = 1, Hindu = 3, no religion = 5 *Postcode*: Participants from 17 different postcodes across four LGAs of Western Sydney. Two postcodes had two participants from each.

Abbreviation: LGA = local government area.

The characteristics of the participants reflect the demographic profile of the multicultural and relatively well‐educated population of Western Sydney compared to Australia (Table 4); however, they had somewhat higher incomes, were older and were more qualified than expected in a Western Sydney population (Table 5).

**Table 4:** Other demographic variables [23].

LGA	Median age	Uni qual%
Parramatta	35	44
Cumberland	34	27
Blacktown	34	29
The Hills	38	40
**Mean Western Sydney population**	**35**	**35**
**Culturally diverse citizens' jury**	**43**	**45**
NSW	39	28
Australia	38	26

*Note:* Bold values indicates direct comparisons of median age and qualifications between the mean Western sydney population and the jury we describe.

**Table 5:** Median income.

Population group	Median income
Greater Sydney [24]	$43,628
Western Sydney [25]	$45,214
Culturally diverse citizens' jury	$62,500

### 3.2 Evidence

A diversity of evidence, experience and perspectives was presented to the jury (Table 6).

**Table 6:** Information sessions.

Content	Presenters with experience/expertise in:	Expert source
*The landscape of health and social care in Western Sydney*: An overview of strengths and challenges including for health, workforce and funding.	Primary health care, dentistry, public health and diabetes	NSW Ministry of Health Western Sydney endocrinologist leader in diabetes prevention WentWest decision‐making Retired Western Sydney dentist and oral health advisor from the Australian Dental Association
*The first 10 years*: The impact of adverse childhood events and the health and social supports, which can prevent and ameliorate childhood trauma	Early childhood and adverse events, maternal and child health, working with families in general practice	Maternal and child health scholar Paediatric general practitioner and clinical leadership with WentWest
*10–20 years*: Homelessness; foster care and the alternatives; mental health and substance abuse; suicide prevention	Trauma, youth health and well‐being, out‐of‐home youth support	Youth welfare leaders Culturally diverse programs for youth Out‐of‐home care innovator for teenagers
*20–40 years*: Integrated care and living with chronic conditions; social connectedness and loneliness; paramedics, urgent care and mental health supports; language barriers	Ambulance, paramedics, pharmacy, connecting services and mental health	Trauma care—NSW Ambulance—paramedicine WentWest program leader Mental health program leader
*40–60 years*: Team approach to primary care; use of data to support patient care; community circles	Disability, aged care, community circles, best practice in integrated primary health care and models of care	General practice leaders/academics in Western Sydney Social prescribing leader
*60+ years*: Social isolation and intergenerational programs; men's sheds; aged, dementia and palliative care	Ageing well, end‐of‐life care, including in cross‐cultural settings, health consumer perspectives	Aged care leader Palliative care clinicians Dementia care leader

Abbreviation: NSW = New South Wales.

### 3.3 Jury Recommendations

In the following section, participant comments are coded for anonymity as juror (J) or consumer advisor (CA).

The final presentation of the recommendations (summarised in Table 7) emphasised that Urgent Care Service expansion was considered a ‘quick win’ and was backed by consensus:

… this is number one for a reason: there is a demand for it, anyone who has waited three to four hours in Mount Druitt Hospital for a sprained ankle knows that if something can be treated in Urgent Care, not in the emergency department, we need this. And because it's already existing, the infrastructure is there, we already have staffing … why not use it? Why not expand it?

J13

**Table 7:** Final recommendations from the citizens' jury.

The jury recommended investment in the following areas: 1. *Preventative health* through evidence‐based programs that intervene early to support kids and their families to thrive at school (e.g., programs for families to reduce adverse childhood events, and sustained home visiting by maternal nurses). (This recommendation is woven through most other recommendations that, if actioned, will contribute to preventative health). 2. *Western Sydney community development* a. Community education particularly in schools for disease prevention and personal supports. b. Develop community hubs as supportive health environments (e.g., to host outreach clinics in hearing and oral health, as places where people can connect to reduce loneliness and find services that support those who do not visit a general practice). c. Co‐design community development approaches with the community including communication channels to share knowledge about how to prevent poor health outcomes. This includes broadcasting widely the *assets* of Western Sydney, including the childhood dental scheme, urgent care centres and primary care pathways and strategies to prevent poor health accessed through a GP. 3. *Focus on team‐based care* that incorporates: a. Allied health (e.g., social workers and mental health workers), care navigators, connectors (e.g., social prescribing through circles of support), paramedics who can come to the home (not ambulance) and case workers, b. Support for GPs to use a proactive preventative model of care c. Relationship‐based care to permit individuals and families in crisis to be known and supported throughout the health care system. 4. *Upscale urgent care service* to allow residents to remain out of hospital and avoid hospital emergency departments. 5. *Improved data sharing across care sectors* (including between hospital and GP and within GP) to be more proactive and predictive. 6. *Invest in workforce development of health professionals* by incentivising medical and allied health students to work in Western Sydney and expediting the registration of overseas trained health professionals. 7. *Support Intergenerational programs* to bring children and aged citizens together for the mutual health and well‐being benefit of both groups. 8. *Develop a culture of relationship‐based care*: a. *Investing in systems that support relationship‐based models for teenagers* dependent on out‐of‐home care 9. *Build resilience*: Ensure that funding goes beyond political cycles. 10. *Evaluate the process*: Measure the return on investment of the aforementioned recommendations for Western Sydney.

Abbreviation: GP = general practice.

In considering the future of health service provision in Western Sydney, all participants supported a focus on prevention. Specifically, investments in early childhood‐linked care must include families, and to prevent the trajectory of diabetes complications communities must be better informed. Similarly, prevent the chronic disease consequences of poor oral health.

… just an oral example. Now, an abscess in the mouth is easily preventable, it's caused by possibly dental caries … that's why prevention is very important, because there is a lot of chronic conditions. Most of them can be, in oral health … there are a lot of preventable conditions that can use resources that we have in our hospitals and other services …

J14

Although much of this focus was on measures that would be considered beyond the remit of a PHN, including national government policy approaches such as subsidies for dental check‐ups and healthy food, the jury recommended that WentWest build coalitions with relevant agencies and trusted community organisations to broaden the audience and strengthen opportunities to enable these recommendations. A coalition could support the development of healthy environments in Western Sydney:

I think it is really important to look at how healthcare impacts other areas of our society. So, for example, with diabetes, our area is so high at risk for having a lot of people with diabetes, so if we improve the accessibility to fresher foods and if we improve the amount of exercise areas that we can have, that are more accessible to people, I think that would be very beneficial …

J20

A coalition could advocate for more health education in schools, sporting clubs and social or religious organisations as well as improved outdoor exercise opportunities and meeting places for youth. Several participants called for empowering individuals to manage their own health and thereby ‘create a community where it is pro‐health’ (J6). Participants called for more outreach and mobile testing including early detection of diabetes and cervical cancer and support for education on oral health.

As part of this approach, the jury recognised the importance of building social cohesion and community in Western Sydney. Participants suggested:

“One of the biggest pros of Western Sydney is that it is indeed a melting hotpot of different cultures, but … sometimes cultures … only want to mingle with each other. We need to reduce that. One way we can do that is investing into community centres, religious places”. (and) “community mentorship leadership programs”.

J18

The citizens' jury and similar events hosted by WentWest were seen as one way to overcome these boundaries, but the jury also called for a formalisation of community development as a core business for the PHN, as one participant explained:

If you are looking at any successful organisation, they have a specific team that looks at corporate social responsibilities. I think that we, as a community, and also integrating with councils, need to work on integrating that into the business practice and the business models that GPs function under.

J18

An integrated holistic approach with an emphasis on continuity of care extended to the type of workforce recommended by the jury. Participants called for a team‐based approach to primary care with the inclusion of nurses, social workers, care navigators, case workers and allied health professionals supporting GPs in the provision of wrap‐around care. The jury believed that this would alleviate the burden on the limited number of GPs available and increase the provision of personalised care, community conversations and people's ability to understand the health system.

Increasing the number and hours of Urgent Care Service Centres [26] in Western Sydney was also seen as a way of alleviating the burden in tertiary settings and bridging the care gap between primary and tertiary organisations. Some participants supported the idea of using an algorithm to initially funnel individual cases into appropriate care:

… you call and an algorithm decides, okay, you need a paramedic or you need a GP or you need a specialist ‐ what you need… So it kind of helps that decision tree and that diagnosis tree and funnelling where the calls need to go. That can make the process of getting into the system a little more efficient and more productive.

J14

Other participants were concerned about excluding people who were technophobes but also in managing community expectations and potential negative community responses to such an approach:

You will get someone who is likely to say, “No‐one answered my call, no‐one came to see me, I had to use this app” which did or didn't work sometimes, “I didn't get the right care, there was no personal touch”.

J1

Several participants were concerned that even where services, such as Urgent Care Service Centres, existed, they were not well‐publicised, and many people were unaware of their existence. Increasing awareness, extending opening hours and opening more Urgent Care Service Centres were all seen as ‘quick wins’ and potential cost‐saving approaches in health care provision for Western Sydney.

An alternative model was the ‘one‐stop shop’. Urgent Care was one such version so that when a person presented to an Urgent Care Service Centre, other items of service might be connected with that person. The idea of connected or integrated care was seen as particularly desirable by the jury with many participants describing issues associated with fragmented care or with transmission of relevant and valuable information between sectors or services. The jury observed that sharing health information across points of contact in the health system was currently problematic. However, although there was support for investment in increased interoperability and data sharing between services, they also indicated that there were no simple solutions. Participants pointed to potential problems with cybersecurity, stability of websites, access, and unknowns in the use of artificial intelligence and automated systems. One participant suggested that certain groups in the community might be particularly vulnerable to issues with data security:

You are talking about technology and medical treatments. I believe that that's leaving the aged out of it again, you know? When it comes to a data breach and stuff, they wouldn't understand how to protect themselves.

J8

The jury indicated that effective communication strategies are essential for empowering people to manage their own health, building community trust and in managing community expectations. A common theme was the contention that many people in the community were unaware of existing services and that more effort was needed to disseminate this information. One participant suggested that transparency around how the PHN uses funds would also help to build trust.

Finally, the jury recommended investment in a range of innovative approaches including social prescribing [27], community circles [28], professional individualised care for troubled youth [29] and intergenerational programs with colocation of aged and child care [30]. All were seen as ways of building community resilience and ameliorating loneliness. At the same time, some jurors called for the generation of evidence to examine the effectiveness and return on investment for such programs in the local setting.

### 3.4 Research Team Consumer Advisor Experiences

The four consumer advisors in our research team yielded insights and added significant value during discussions. The group reported initial uncertainty (‘not sure what was expected of me’ (CA1), ‘somewhat intimidated at first’ (CA2)) but growth of confidence over time (‘finding my feet … more confident to contribute’ (CA2), ‘I felt safe amongst you all’ (CA1)) and overall a positive experience (‘very educational and worthwhile’ (CA3), ‘my input was valued and it enriched the experience for me’ (CA2)).

## 4 Conclusions

The value of involving citizens in those decisions which directly affect them and which involve publicly funded services is widely recognised [31]. Citizen participation in decision‐making acts to build trust, accountability and integrity and improve the delivery of public services to better meet the needs of a population. Public engagement occurs on a spectrum of participation from town hall meetings through to survey or focus group research and on to more formal deliberative inclusive methods such as citizens' juries and deliberative polling. Deliberative inclusive methods are useful for ensuring that the engagement is informed both by expert evidence and the experience and views of fellow citizens and are particularly valuable in public engagement around complex policy questions.

Deliberative inclusive publics have been used for the development of policy advice in several government sectors in Australia including the development of a cycling policy in South Australia [32], obesity prevention in Victoria [33] and a future vision plan for Sydney [34]. They have also been used in research settings with and without the involvement of a sponsoring organisation with the intent to use the findings [11, 21]. Translation of recommendations directly into policy has always been the most difficult aspect of deliberative democratic publics [11, 12], although uptake is generally better when the process has been initiated by the government. For example, all the recommendations of the South Australian cycling jury were adopted into policy and practice.

The use of a deliberative inclusive public to review informed public perspectives on priority setting in primary care settings is uncommon. Kashefi and Mort (2004) reported a citizens' jury used by an English Primary Care Group to determine public views on ‘What would improve the health and well‐being of residents’ [35]. Mooney and Blackwell [36] conducted two juries in Western Australia: one looking to elicit broad public values that might be used to support budgetary decision‐making in primary care, and the other looked more closely at measures to reduce inequity. We note that a recent systematic review of citizens' juries focused on health policy generally rather than primary care specifically [11].

Our culturally diverse jury was asked a very broad question, motivated by the desire of the PHN to understand the basic preferences in health and social care from the point of view of its citizens. The breadth of the question required presenting a wide range of evidence with limited opportunity for digression into specific related topics. An example was ‘homelessness’, which was suggested as the topic for a separate jury:

(homelessness) is such a big factor that affects health. We know that it is not going to be done, it is a big chunk, but at least it needs to be in the discussion, because it affects health very much.

J1/13

The exercise described in this article was the first example of the use of a multicultural and diverse, informed and deliberative citizens' jury to be engaged by an Australian PHN to advise on future directions for regional health care. The jury brought together a diverse group of people with broad experience and cultural backgrounds, in a facilitated and respectful manner, to deliberate on an issue of major importance to the region. Limiting the recruitment to over 18 years meant that our median age was skewed away from the young population in Western Sydney; however, a future youth jury may be reasonable especially given the jury recommendation that intervening early in life for better health and well‐being outcomes was unanimous.

The recommendations were well received by WSPHN, and the organisation has undertaken to establish a permanent citizens' assembly with rolling membership to advise the organisation on an ongoing basis, similar to the Ostbelgian model [37] that monitors all recommendations of all juries in their region. Since the jury in August 2023, there were five meetings with participants to co‐design the scope and terms of reference and to achieve the modelling required for permanency. WSPHN has committed to funding the model and looking for Western Sydney coalition partners who can work with the PHN to realise the recommendations made by the jury. The findings were also presented to State politicians and further follow‐up will be made with relevant Ministers for education, housing, justice and immigration to work on the jury's recommendations. The recommendations are also being shared with local organisations including local and state government departments and health and social care providers to join with WentWest.

This research suggests that deliberative inclusive methods such as diverse deliberative citizen juries can be useful tools for effective public engagement in a primary health care setting, particularly in the diverse and complex PHNs used in Australia.

## Author Contributions

Coralie Wales (Western Sydney University) designed the study, coordinated and conducted the study, facilitated the citizens' jury, analysed the data, revised the draft manuscript and approved the final manuscript. Penny Abbott (Western Sydney University) designed the study, contributed to the citizens' jury, analysed the data, revised the manuscript and approved the final manuscript. Jackie Street (University of Adelaide) provided guidance within the research team, analysed data, drafted the initial manuscript and approved the final manuscript. Lauren Fawcett (WentWest Relationships to Partnerships Practitioner) coordinated and conducted the study, contributed to the citizens' jury, analysed data, contributed to revisions and approved the final manuscript. Maree Jennings (Aboriginal and Torres Strait Islander facilitator) and Philip Lee (WentWest Consumer Advisory Councillor) contributed to the citizens' jury, contributed to the analysis of the data, contributed to revisions and approved the final manuscript. Wendy Sharp (WentWest Consumer Advisor) contributed to the analysis of data, contributed to revisions and approved the final manuscript. All authors contributed to the discussions.

## Ethics Statement

This research was approved by the Western Sydney University Human Research Ethics Committee (HREC approval H15450).

## Consent

There were no patients involved in this research; however, four volunteer consumer representatives and advisors were recruited to the research team. They consented to participate and to being included in the ethics submissions.

## Conflicts of Interest

The authors declare no conflicts of interest.

## Data Availability

The data that support the findings of this study are available from the corresponding author upon reasonable request.
